# ﻿Morphology and multigene phylogeny reveals five new species of Hydnaceae (Cantharellales, Basidiomycota) from China

**DOI:** 10.3897/mycokeys.119.154387

**Published:** 2025-06-26

**Authors:** Qian Zhou, Chengbin Qian, Chuyun Zhang, Qidong Su, Yiliang Li, Shihui Zhang, Nian Mu, Taimin Xu, Hongmin Zhou, Changlin Zhao

**Affiliations:** 1 The Key Laboratory of Forest Resources Conservation and Utilization in the Southwest Mountains of China Ministry of Education, Key Laboratory of National Forestry and Grassland Administration on Biodiversity Conservation in Southwest China, Yunnan Provincial Key Laboratory for Conservation and Utilization of In-forest Resource, Southwest Forestry University, Kunming 650224, China; 2 College of Forestry, Southwest Forestry University, Kunming 650224, China; 3 Yunnan Tongbiguan Provincial Nature Reserve, Mangshi 679319, China; 4 Yunnan Key Laboratory of Gastrodia and Fungal Symbiotic Biology, Zhaotong University, Zhaotong, 657000, China

**Keywords:** Asia, biodiversity, molecular systematics, taxonomy, wood-inhabiting fungi

## Abstract

Wood-inhabiting fungi play a fundamental role in ecosystem processes, particularly in wood degradation and the recycling of organic matter. In this study, a new genus, *Clavuliella***gen. nov.**, and five new species, viz. *Burgellaalbofarinacea***sp. nov.**, *B.fissurata***sp. nov.**, *Burgoawumengshanensis***sp. nov.**, *Clavuliellasinensis***sp. nov.**, and *Sistotremasinense***sp. nov.**, are described from China and illustrated based on morphological characteristics and molecular phylogenetic analyses. Sequences of the ITS+nLSU genes were used for the phylogenetic analyses using Maximum Likelihood, Maximum Parsimony, and Bayesian Inference methods. The phylogram of the family Hydnaceae, based on the ITS+nLSU rDNA gene regions, included four genera; *Burgella*, *Burgoa*, *Clavuliella* and *Sistotrema.* The topology based on these sequences revealed that *Burgellaalbofarinacea* was closely related to *B.flavoparmeliae*, and *B.fissurata* was grouped with *B.lutea*. The taxon *Burgoawumengshanensis* was sister to the clade that included *B.anomala* and *B.verzuoliana*. The species *Sistotremasinense* was grouped closely with *S.brinkmannii* and *S.farinaceum*. All new taxa can be readily recognized by their macroscopic and anatomical characteristics. The five new species, closely related taxa in the phylogenetic tree, and morphologically similar species are discussed.

## ﻿Introduction

Fungi constitute an integral and valuable part of our natural ecosystem and play an essential ecological role in driving carbon cycling in forest soils, mediating mineral nutrition of plants, and alleviating carbon limitations (Chen et al. 2023a, b; Niego et al. 2023; Dong et al. 2024a; Yang et al. 2025). The wood-inhabiting fungal family Hydnaceae Chevall includes many variations of the fruiting body types within the order Cantharellales J. Schröt (Cai and Zhao 2023; Gao et al. 2024), in which it comprises many representative wood-inhabiting fungi taxa, such as bulbil-shaped, hypochnoid, corticioid, odontoid, poroid, clavarioid, ramarioid, mucronelloid, cantharelloid, and hydnoid basidiomes with diverse hymenophoral and cystidial morphology (Uehling et al. 2012; Diederich et al. 2014; Gruhn et al. 2017; Masumoto and Degawa (2020); Lawrey et al. 2020; Bondartseva and Zmitrovich 2023; Cai and Zhao 2023; Gao et al. 2024).

The genus *Burgella* Diederich & Lawrey (Hydnaceae, Cantharellales), typified by *B.flavoparmeliae* Diederich & Lawrey, is characterized by the following features: conidia cylindrical, conidiophores short, hyphae hyaline, septate, straight, rarely branched or anastomosed. Agglomerations of bulbils gelatinous in appearance, almost coralloid, composed of irregularly shaped bulbils; bulbils externally and internally composed of irregular, roundish or elongate cells with clamped septa (Diederich and Lawrey 2007). Based on the MycoBank database (http://www.mycobank.org, accessed on 30 March 2025) and the Index Fungorum (http://www.indexfungorum.org, accessed on 30 March 2025), the genus *Burgella* has 2 registered species and intraspecies names (Diederich and Lawrey 2007). Previous studies have shown that *B.flavoparmeliae* and *Sistotremaoblongisporum* M.P. Christ. & Hauerslev were the sister group of the genus *Multiclavula* (Diederich and Lawrey 2007). The species *B.flavoparmeliae* was only distantly related to the type species of the genus *Burgoa* Goid., which appeared in a different place in the order Cantharellales, and the research revealed that *B.flavoparmeliae* should not be included in *Burgoa*, but instead placed in the new genus *Burgella* (Diederich and Lawrey 2007).

The genus *Burgoa*, typified by *B.verzuoliana* Goid. (Hydnaceae, Cantharellales, Agaricomycotina), was established by Goidŕnich to accommodate microfungi producing multicellular spore-like structures with differentiated peridial and internal cells, i.e. bulbils. Apart from the production of the bulbils, members of this genus were distinguished by the formation of clamp connections on their mycelium. This feature showed their affinity to members of Agaricomycotina, but their position within the order Cantharellales was recognised only recently (Diederich and Lawrey 2007; Koukol and Kubátová 2015; Kiyuna et al. 2015). The genus *Burgoa* is a peculiar microscopic basidiomycete not forming any basidiocarps in its life cycle. So far, this saprotroph has sporadically been found mainly on different woody substrates but the overall knowledge of its ecology and distribution remains sparse due to its rarity (Koukol and Kubátová 2015). Based on the MycoBank database (http://www.mycobank.org, accessed on 30 March 2025) and the Index Fungorum (http://www.indexfungorum.org, accessed on 30 March 2025), the genus *Burgoa* has 10 registered species and intraspecies names (Diederich and Lawrey 2007; Koukol and Kubátová 2015; Kiyuna et al. 2015).

*Clavulina* J. Schröt. (Hydnaceae, Cantharellales), with *Clavulinacristata* (Holmsk.) J. Schröt. as its type species, was established in 1888 (Schröter 1888; He et al. 2019). In China, 14 *Clavulina* species have been reported on the basis of morphological and molecular analyses, most of which are found in subtropical regions (Gao et al. 2024). In the present study, the new genus *Clavuliella* falls within Hydnaceae (Cantharellales) and is closely related to *Clavulina*.

The genus *Sistotrema* Fr. (Hydnaceae, Cantharellales, Agaricomycetes, Agaricomycotina, Basidiomycota), typified by *S.confluens* Pers., is a comparatively large genus belonging to the phylum Basidiomycota and is morphologically characterized by resupinate or pileate-stipitate, soft basidiomes, smooth, grandinioid, hydnoid, or poroid hymenophore with various characteristic textures (pellicular, membranaceous, or ceraceous), a monomitic hyphal system with oily inclusions, urniform basidia, and smooth, thin-walled, basidiospores containing cytoplasmic oil droplets (Eriksson et al. 1984; Bernicchia and Gorjón 2010; Cai and Zhao 2023). Based on the MycoBank database (http://www.mycobank.org, accessed on 30 March 2025) and the Index Fungorum (http://www.indexfungorum.org, accessed 30 March 2025), the genus *Sistotrema* has 224 registered species and intraspecies names, however the actual number of recognized species is 111 (Eriksson et al. 1984; Bernicchia and Gorjón 2010; Sugawara et al. 2022; Cai and Zhao 2023).

In the present study, extensive morphological examinations, combined with analyses of multi-gene sequences data, support the introduction of a new genus and 4 new species of wood-inhabiting fungi. Descriptions and illustrations based on morphological characteristics are presented along with evidence from phylogenetic analyses.

## ﻿Materials and methods

### ﻿Morphology

Fresh basidiomata of the fungi growing on angiosperm branches were collected from the Dali, Dehong, and Zhaotong of Yunnan Province, and Guiyang of Guizhou Province, P.R. China. Specimens were dried in an electric food dehydrator at 40 °C (Dong et al. 2024b) then sealed and stored in an envelope and deposited in the Herbarium of the Southwest Forestry University (SWFC), Kunming, Yunnan Province, P.R. China. Macromorphological descriptions were based on field notes and photos captured in the field and lab. Colour terminology followed Petersen (1996). Micromorphological data were obtained from the dried specimens when observed under a light microscope following the previous study (Cai and Zhao 2023). The following abbreviations are used for the micro characteristic description: KOH = 5% potassium hydroxide water solution, CB = Cotton Blue, CB– = acyanophilous, IKI = Melzer’s Reagent, IKI– = both inamyloid and indextrinoid, L = mean spore length (arithmetic average for all spores), W = mean spore width (arithmetic average for all spores), Q = variation in the L/W ratios between the specimens studied and n = a/b (number of spores (a) measured from given number (b) of specimens).

### ﻿Molecular phylogeny

The EZNA HP Fungal DNA Kit (Omega Biotechnologies Co., Ltd., Kunming, China) was used to extract DNA with some modifications from the dried specimens. The nuclear ribosomal ITS region was amplified with primers ITS5 and ITS4 (White et al. 1990). The PCR procedure for ITS was as follows: initial denaturation at 95 °C for 3 min, followed by 35 cycles at 94 °C for 40 s, 58 °C for 45 s and 72 °C for 1 min, with a final extension of 72 °C for 10 mins. The nuclear nLSU region was amplified with primer pair LR0R and LR7 (Rehner and Samuels 1994). The PCR procedure for nLSU was as follows: initial denaturation at 94 °C for 1 min, followed by 35 cycles at 94 °C for 30 s, 48 °C for 1 min and 72 °C for 1.5 mins with a final extension of 72 °C for 10 mins. The PCR procedure for ITS and nLSU followed a previous study (Zhao and Wu 2017). All newly generated sequences were deposited in NCBI GenBank (https://www.ncbi.nlm.nih.gov/genbank/) (Table 1).

**Table 1.** Names, specimen numbers, references and corresponding GenBank accession numbers of the taxa used in this study.

Species name	Specimen No.	GenBank accession No.		Country	References
		ITS	nLSU		
*Bergerellaatrofusca*	BR Berger 34240	MN902070	MN902070	Austria	Lawrey et al. (2020)
*Bryoclavulaphycophila*	Hiroshi:Bryoclavula4	OQ791465	OQ791464	Japan	NCBI Database
*B.phycophila*	S-287-FB3	LC544109	—	Japan	Masumoto and Degawa (2020)
***Burgellaalbofarinacea***	**CLZhao 31820**	**PQ758751**	**PQ758759**	**China**	**Present study**
***B.albofarinacea***	**CLZhao 32468**	**PQ758754**	**PQ758762**	**China**	**Present study**
***B.albofarinacea***	**CLZhao 32026**	**PQ758753**	**PQ758761**	**China**	**Present study**
***B.albofarinacea***	**CLZhao 31855**	**PQ758752**	**PQ758760**	**China**	**Present study**
***B.fissurata***	**CLZhao 30212**	**PQ758749**	**PQ758757**	**China**	**Present study**
*B.flavoparmeliae*	Flakus 23513	—	KC336074	USA	Diederich et al. (2014)
*B.flavoparmeliae*	Buck 38682	—	DQ915469	Bolivia	Diederich et al. (2014)
*B.flavoparmeliae*	JL192-01 SV1	OR471304	—	USA	Swenie et al. (2023)
*B.flavoparmeliae*	JL192-01 SV2	OR471305	—	USA	Swenie et al. (2023)
*B.flavoparmeliae*	JL192-01 SV3	OR471306	—	USA	Swenie et al. (2023)
*B.flavoparmeliae*	JL192-01 SV4	OR471307	—	USA	Swenie et al. (2023)
*B.lutea*	Etayo 27623	KC336076	KC336075	Bolivia	Diederich et al. (2014)
*Burgella* sp.	WS34_1_2_A_As_10000	LC631658	—	Japan	Unpublished
*Burgella* sp.	HHB-19354	MW740322	—	New Zealand	Unpublished
*Burgella* sp.	HHB-19352	MW740323	—	New Zealand	Unpublished
*Burgoaanomala*	CBS 130.38	AB972780	—	Japan	Kiyuna et al. (2015)
***B.wumengshanensis***	**CLZhao 33227**	**PQ758755**	—	**China**	**Present study**
*B.verzuoliana*	CBS 131.38	AB972781	—	Italy	Kiyuna et al. (2015)
***Clavuliellasinensis***	**CLZhao 31231**	**PQ758750**	**PQ758758**	**China**	**Present study**
*Clavulinacristata*	EL95_97	—	AY586648	Sweden	Larsson et al. (2004)
*C.iris*	ML 5135C1	MN028412	MN028396	Cyprus	Campo et al. (2023)
*C.minor*	B30912949	OP738993	OP737360	China	Gao et al. (2024)
*C.minor*	B30912949	OR149156	OR145333	China	Gao et al. (2024)
*C.parvispora*	FCME 27650	MH542550	MN049492	Mexico	Gao et al. (2024)
*C.parvispora*	FCME 27657	MH542549	MN049491	Mexico	Gao et al. (2024)
*C.samuelsii*	TENN065723	JQ638712	—	USA	Gao et al. (2024)
*C.samuelsii*	PDD:89881	GU222317	—	New Zealand	Gao et al. (2024)
*C.subrugosa*	TENN043395	JQ638711	—	USA	Gao et al. (2024)
*C.subrugosa*	TN43395	JN228221	JN228221	New Zealand	Gao et al. (2024)
*C.sphaeropedunculata*	FCME 27661	MH542560	MK253716	Mexico	Gao et al. (2024)
*C.sphaeropedunculata*	MEXU 28222	MH542557	MK253717	Mexico	Gao et al. (2024)
*Hydnumalbidum*	MB11-6024/2	—	AY293186	Thailand	Binder et al. (2005)
*H.albomagnum*	AFTOL-ID 471	DQ218305	AY700199	USA	Masumoto and Degawa (2020)
*H.rufescens*	MB18-6024/1	—	AY293187	Panama	Binder et al. (2005)
*Minimedusaobcoronata*	CBS 120605	GQ303278	GQ303309	USA	Diederich and Lawrey (2007)
*M.polyspora*	CBS:113.16	—	MH866167	USA	Vu et al. (2019)
*M.polyspora*	SH-Ecto-3	—	MG833798	China	NCBI Database
*Multiclavulacaput-serpentis*	KaiR699	MW386064	MW369074	Japan	Reschke et al. (2021)
*M.corynoides*	Lutzoni 930804-2	U66440	U66440	USA	Lutzoni (1997)
*M.mucida*	AFTOL-ID 1130	DQ521417	AY885163	Switzerland	Masumoto and Degawa (2020)
*M.petricola*	NBRC 114399	LC516464	LC516465	USA	Masumoto and Degawa (2020)
*M.vernalis*	Lutzoni 930806-1	U66439	U66439	USA	Lutzoni (1997)
*Neoburgoafreyi*	JL596-16	KX423755	KX423755	Vietnam	Lawrey et al. (2016)
*N.freyi*	EZ4455	OR471314	OR471068	Canada	Swenie et al. (2023)
*Platygloeadisciformis*	AFTOL-ID 710	DQ234556	AY629314	USA	Sugawara et al. (2022)
*Rogersiomycesmalaysianus*	LE-BIN 3507	KT779285	—	Poland	Psurtseva et al. (2016)
*Sistotremaconfluens*	FCUG 298	—	DQ898711	Canada	Moncalvo et al. (2006)
*S.confluens*	AFTOL-ID 613	DQ267125	AY647214	Canada	Masumoto and Degawa (2020)
*S.adnatum*	FCUG 700	—	DQ898699	Sweden	Moncalvo et al. (2006)
*S.adnatum*	GB700	OR464426	OR460895	Sweden	Swenie et al. (2023)
*S.alboluteum*	TAA167982	—	AY586713	Canada	Larsson et al. (2004)
*S.alboluteum*	TAA180259	—	AJ606042	Sweden	Nilsson et al. (2006)
*S.albopallescens*	KHL11070	—	AM259210	Canada	Nilsson et al. (2006)
*S.athelioides*	FCUG 701	—	DQ898700	Japan	Moncalvo et al. (2006)
*S.brinkmannii*	NH11412	—	AF506473	Sweden	Larsson et al. (2004)
*S.biggsiae*	FCUG 782	—	DQ898697	Sweden	Moncalvo et al. (2006)
*S.chloroporum*	TUMH 64399	NR178117	LC642057	Sweden	Sugawara et al. (2022)
*S.citriforme*	KHL15898	KF218962	KF218962	Sweden	Kotiranta and Larsson (2013)
*S.coroniferum*	GB-BN-2	—	AM259215	Canada	Nilsson et al. (2006)
*S.coroniferum*	KH Larsson s.n.	KF218968	KF218968	Netherlands	Kotiranta and Larsson (2013)
*S.coronilla*	NH7598	—	AF506475	USA	Larsson et al. (2004)
*S.efibulatum*	FCUG 1175	—	DQ898696	Canada	Moncalvo et al. (2006)
*S.epiphyllum*	CBS H-21517	NR155795	—	Canada	NCBI Database
*S.eximum*	Thorn429	—	AF393076	Finland	Binder and Hibbett (2002)
*S.eximum*	CBS:531.91	MH862275	MH873956	Japan	Vu et al. (2019)
*S.farinaceum*	FCUG 659	—	DQ898707	Japan	Moncalvo et al. (2006)
*S.farinaceum*	HK23176	KF218963	KF218963	Australia	Kotiranta and Larsson (2013)
*S.flavorhizomorphae*	TUMH:64401	LC642038	LC642059	Finland	Sugawara et al. (2022)
*S.flavorhizomorphae*	TUMH:64402	LC642040	LC642060	Sweden	Sugawara et al. (2022)
*S.hypogaeum*	CBS 394.63	MH858314	MH869926	Finland	Vu et al. (2019)
*S.luteoviride*	H HK23176	NR158892	—	Sweden	Kotiranta and Larsson (2013)
*S.muscicola*	KHL8791	—	AF506474	Canada	Larsson et al. (2004)
*S.muscicola*	KHL 11721	—	AJ606040	USA	Nilsson et al. (2006)
*S.oblongisporum*	KHL 14077	—	KF218970	Spain	Kotiranta and Larsson (2013)
*S.octosporum*	FCUG 2822	—	DQ898698	USA	Moncalvo et al. (2006)
*S.octosporum*	CBS:126038	MH864053	MH875510	Finland	Vu et al. (2019)
*S.pistilliferum*	EL 28/10	KF218964	KF218964	Canada	Kotiranta and Larsson (2013)
*S.raduloides*	AFTOL-ID 619	—	AY647213	Sweden	Masumoto and Degawa (2020)
*S.raduloides*	LR 44004	KF218969	KF218969	USA	Kotiranta and Larsson (2013)
*S.resinicystidium*	FCUG 2188	—	DQ898708	China	Moncalvo et al. (2006)
*S.sernanderi*	GB-BN-4	—	AM259219	China	Nilsson et al. (2006)
*S.sernanderi*	PUL:F24593	MW448599	—	China	NCBI Database
***S.sinense***	**CLZhao 24876**	**PQ758748**	**PQ758756**	**China**	**Present study**
*S.subconfluens*	Dai 12577	JX076812	JX076810	China	Zhou and Qin (2013)
*S.subconfluens*	Dai 12578	—	JX076811	Sweden	Zhou and Qin (2013)
*S.yunnanense*	CLZhao 7357	ON817194	ON810362	USA	Cai and Zhao (2023)
*S.yunnanense*	CLZhao 7395	ON817195	ON810363	UK	Cai and Zhao (2023)
*S.brinkmannii*	NH11412	—	AF506473	Thailand	Larsson et al. (2004)
*Sistotremellaperpusilla*	CBS 126048	MH864061	MH875516	USA	Vu et al. (2019)
*S.perpusilla*	HFRG EJ210404	OL828790	—	Panama	NCBI Database

**Figure 1.**
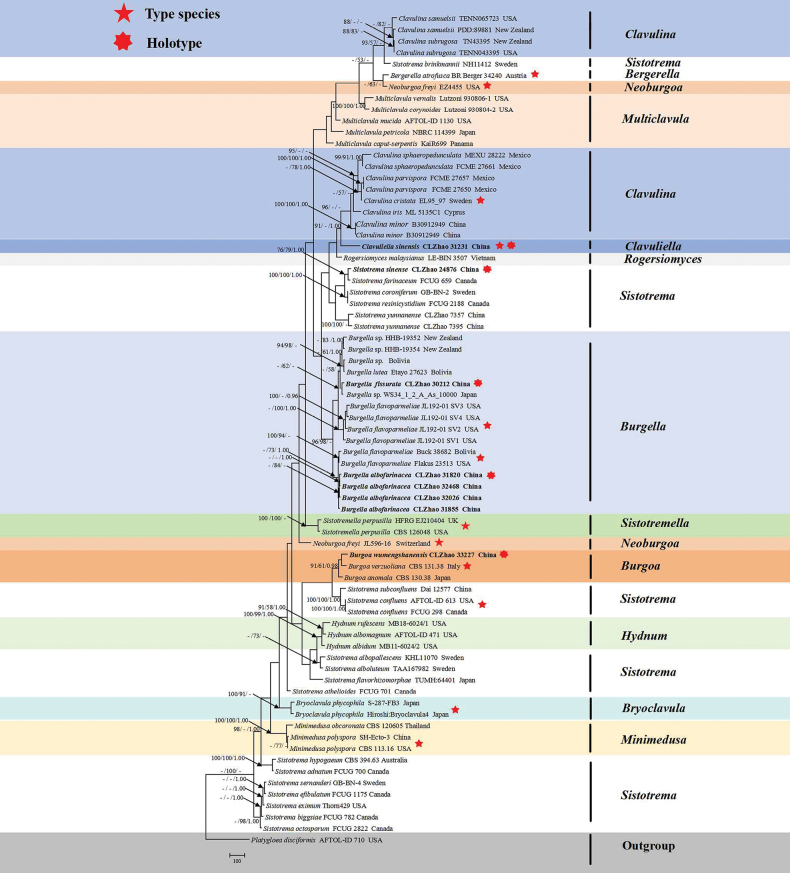
Maximum Parsimony strict consensus tree illustrating the phylogeny of four new species and a new genus within Hydnaceae, based on ITS+nLSU sequences. Branches are labelled with Maximum Likelihood bootstrap values ≥ 70%, parsimony bootstrap values ≥ 50% and Bayesian posterior probabilities ≥ 0.95, respectively.

**Figure 2.**
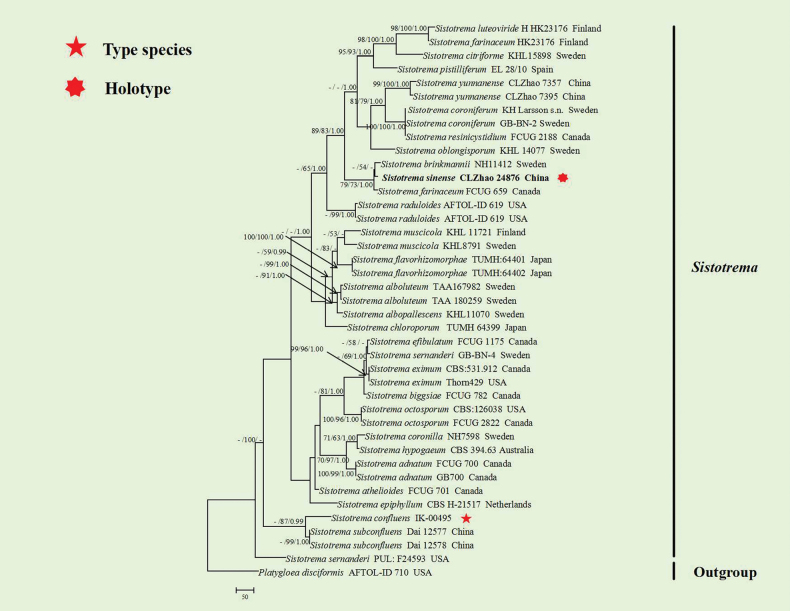
Maximum parsimony strict consensus tree illustrating the phylogeny of the one new species and related species in *Sistotrema*, based on ITS+nLSU sequences. Branches are labelled with Maximum Likelihood bootstrap values > 70%, parsimony bootstrap values > 50% and Bayesian posterior probabilities > 0.95, respectively.

The sequences were aligned in MAFFT version 7 (Katoh et al. 2019) using the G-INS-i strategy. The alignment was adjusted manually using AliView version 1.27 (Larsson 2014). The sequence alignments were deposited in TreeBase (https://treebase.org/treebase-web/home.html;jsessionid=4359D218F4D60336C2A9F7EB7D135CCD) (ID 32177 (Fig. 1)). The sequence alignments were deposited in TreeBase (https://treebase.org/treebase-web/home.html;jsessionid=4359D218F4D60336C2A9F7EB7D135CCD) (ID 32178 (Fig. 2)). Sequences of *Platygloeadisciformis* (Fr.) Neuhoff retrieved from GenBank were used as the outgroup in the ITS+nLSU analysis (Figs 1, 2; Sugawara et al. 2022).

Maximum Parsimony (MP), Maximum Likelihood (ML), and Bayesian Inference (BI) analyses were applied to the combined three datasets following a previous study (Zhao and Wu 2017). All characters were equally weighted and gaps were treated as missing data. Trees were inferred using the heuristic search option with TBR branch swapping and 1,000 random sequence additions. Max trees were set to 5,000, branches of zero length were collapsed, and all parsimonious trees were saved. Clade robustness was assessed using bootstrap (BT) analysis with 1,000 pseudo replicates (Felsenstein 1985). Descriptive tree statistics - tree length (TL), composite consistency index (CI), composite retention index (RI), composite rescaled consistency index (RC) and composite homoplasy index (HI) – were calculated for each maximum parsimonious tree generated. The combined dataset was also analysed using Maximum Likelihood (ML) in RAxML-HPC2 through the CIPRES Science Gateway (Miller et al. 2012). Branch support (BS) for the ML analysis was determined by 1,000 bootstrap pseudo replicates.

MrModelTest 2.3 (Nylander 2004) was used to determine the best-ﬁt evolution model for each dataset for the purposes of Bayesian Inference (BI) which was performed using MrBayes 3.2.7a with a GTR+I+G model of DNA substitution and a gamma distribution rate variation across sites (Ronquist et al. 2012). A total of four Markov chains were run for two runs from random starting trees for 2 million generations for ITS+nLSU (Fig. 1) and 2 million generations for ITS+nLSU (Fig. 2) with trees and parameters sampled every 1,000 generations. The ﬁrst quarter of all the generations were discarded as burn-in. A majority rule consensus tree was computed from the remaining trees. Branches were considered as significantly supported if they received a Maximum Likelihood bootstrap support value (BS) of ≥ 70%, a maximum parsimony bootstrap support value (BT) of ≥ 70%, or a Bayesian posterior probability (BPP) of ≥ 0.95.

## ﻿Results

### ﻿Molecular phylogeny

*Burgellaalbofarinacea* BLASTN homology search using the ITS nucleotide sequence indicated that the sequence had 87% identity with the sequence as OR471304, named *Burgellaflavoparmeliae* from the NCBI culture collection (551/635 bp); the nLSU sequence had 98% identity with the sequence as DQ915469, named *B.flavoparmeliae* from the NCBI culture collection (1294/1323 bp). *Burgellafissurata* BLASTN homology search using the ITS nucleotide sequence indicated that the sequence had 88% identity with the sequence as OR471304, named *B.flavoparmeliae* from the NCBI culture collection (553/627 bp); the nLSU sequence had 98% identity with the sequence as DQ915469, named *B.flavoparmeliae* from the NCBI culture collection (1290/1319 bp). *Burgoawumengshanensis* BLASTN homology search using the ITS nucleotide sequence indicated that the sequence had 83% identity with the sequence as AB972780, named *Burgellaflavoparmeliae* from the CBS culture collection (532/643 bp). *Clavuliellasinensis* BLASTN homology search using the ITS nucleotide sequence indicated that the sequence had 88% identity with the sequence as MT196962, named *Clavulinacastaneipes* (G.F. Atk.) Corner. from the NCBI culture collection (602/688 bp); the nLSU sequence had 96% identity with the sequence as OM942737, named *C.iris* from the NCBI culture collection (1281/1338 bp). *Sistotremasinense* BLASTN homology search using the ITS nucleotide sequence indicated that the sequence had 81% identity with the sequence as OM100765, named *S.coroniferum* (Höhn. & Litsch.) Donk from the NCBI culture collection (541/669 bp); the nLSU sequence had 99% identity with the sequence as OR460882, named *S.brinkmannii* (Bres.) J. Erikss. from the NCBI culture collection (1324/1340 bp).

The ITS+nLSU dataset (Fig. 1) comprised sequences from 55 fungal specimens representing 73 taxa. The dataset had an aligned length of 3499 characters, of which 2000 characters were constant, 546 were variable and parsimony-uninformative, and 953 were parsimony-informative. Maximum parsimony analysis yielded 6 equally parsimonious trees (TL = 5747, CI = 0.4047, HI = 0.5953, RI = 0.5604 and RC = 0.2268). The best model of nucleotide evolution for the ITS+nLSU dataset estimated and applied in the Bayesian analysis was found to be GTR+I+G. Bayesian analysis and ML analysis resulted in a similar topology as in the MP analysis. The Bayesian analysis had an average standard deviation of split frequencies = 0.207165 (BI) and the effective sample size (ESS) across the two runs is double the average ESS (avg. ESS) = 362.5. The phylogram, based on the ITS+nLSU rDNA gene regions (Fig. 1), included five genera, viz. *Burgella*, *Burgoa*, *Clavuliella* and *Sistotrema*, within the family Hydnaceae (Cantharellales, Agaricomycetes). The phylogenetic tree (Fig. 1) inferred from the ITS and nLSU sequences highlighted that the two new species were grouped into the genus *Burgella*, in which *B.albofarinacea* was closely related to *B.flavoparmeliae*, and *B.fissurata* was grouped with *B.lutea* Diederich, Capdet, A.I. Romero & Etayo. The phylogram based on the ITS and nLSU data (Fig. 1) showed that *Burgoawumengshanensis* was clustered into the genus *Burgoa*, in which it was closely related to *B.anomala* (Hotson) Goid. and *B.verzuoliana* Goid. The phylogram based on the ITS+nLSU rDNA gene regions (Fig. 1), included one new species, viz. *Sistotremasinense*, in which it was grouped into the genus *Sistotrema*.

The ITS+nLSU dataset (Fig. 2) comprised sequences from 30 fungal specimens representing 40 taxa. The dataset had an aligned length of 2031 characters, of which 1085 characters were constant, 365 were variable and parsimony-uninformative, and 581 were parsimony-informative. Maximum parsimony analysis yielded 12 equally parsimonious trees (TL = 2690, CI = 0.5487, HI = 0.4513, RI = 0.5550 and RC = 0.3045). The best model of nucleotide evolution for the ITS+nLSU dataset estimated and applied in the Bayesian analysis was found to be GTR+I+G. Bayesian analysis and ML analysis resulted in a similar topology as in the MP analysis. The Bayesian analysis had an average standard deviation of split frequencies = 0.005023 (BI) and the effective sample size (ESS) across the two runs is double the average ESS (avg. ESS) = 813.5. The phylogenetic tree (Fig. 2), inferred from the ITS+nLSU sequences, highlighted that *Sistotremasinense* was grouped closely with *S.brinkmannii* (Bres.) J. Erikss. and *S.farinaceum* Hallenb.

### ﻿Taxonomy

#### ﻿ Burgella albofarinacea

Taxon classificationFungiCantharellalesHydnaceae

Q. Zhou & C.L. Zhao sp. nov.

6A972891-96CF-54B2-8095-7E6FA2D87CF4

857296

Figs 3 , 4 , 5

##### Holotype.

China • Yunnan Province, Zhaotong, Yiliang County, Longhai Town, Jianfeng mountain, GPS coordinates: 27°76′N, 104°37′E, altitude: 1777 m asl., on the fallen branch of angiosperm, leg. C.L. Zhao, 26 August 2023, CL Zhao 31820 (SWFC).

##### Etymology.

*albofarinacea* (Lat.): refers to the albicans and farinaceous hymenophore of the type specimens.

##### Description.

Basidiomata annual, resupinate, adnate, pellicular, coriaceous, without odor or taste when fresh, up to 11.5 cm long, 2 cm wide, 50–100 µm thick. Hymenial surface smooth, farinaceous, white when fresh and drying, cracked. Sterile margin thin, white, thinning out, up to 1 mm wide.

**Figure 3.**
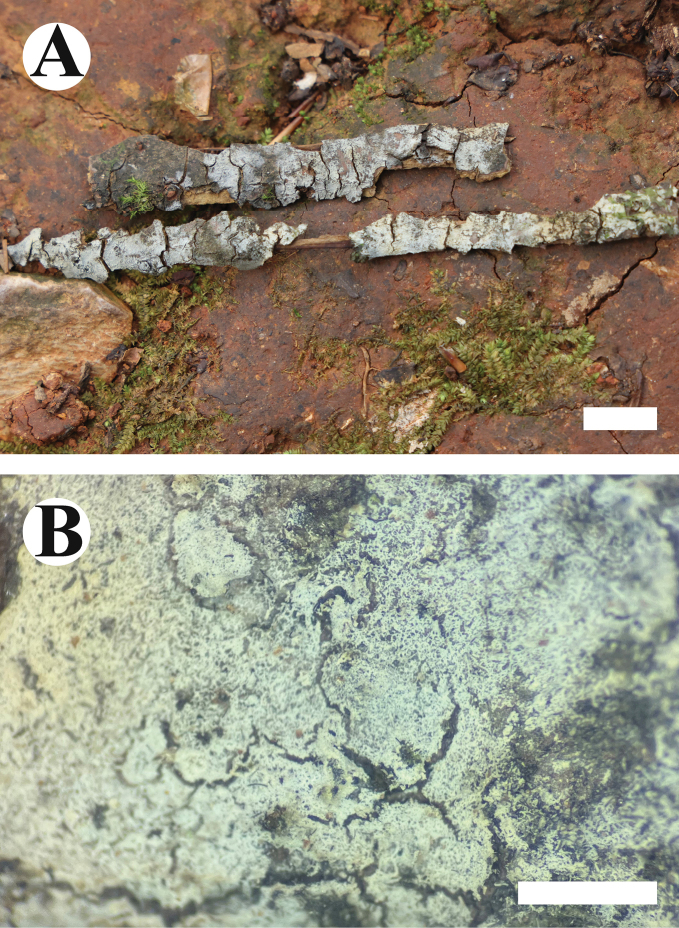
Basidiomata of *Burgellaalbofarinacea* (holotype). Scale bars: 1 cm (**A**); 1 mm (**B**).

Hyphal system monomitic, generative hyphae with clamp connections, sometimes with oily contents, colorless, slightly thick-walled, frequently branched, interwoven, 3.5–5.5 μm in diameter; IKI–, CB–, tissues unchanged in KOH.

**Figure 4.**
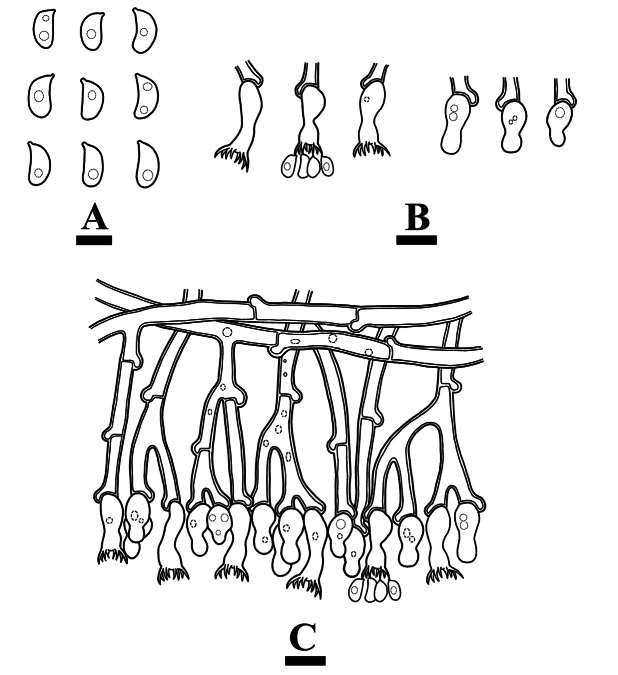
Microscopic structures of *Burgellaalbofarinacea* (holotype): basidiospores (**A**), basidia and basidioles (**B**), a section of hymenium (**C**). Scale bars: 5 µm (**A**); 10 µm (**B, C**).

Cystidia and cystidioles absent. Basidia suburniform to urniform, slightly thick-walled, with 8 sterigmata and a basal clamp connection, 10.5–22 × 3.5–7 μm; basidioles abundant, in shape similar to basidia, but slightly smaller.

**Figure 5.**
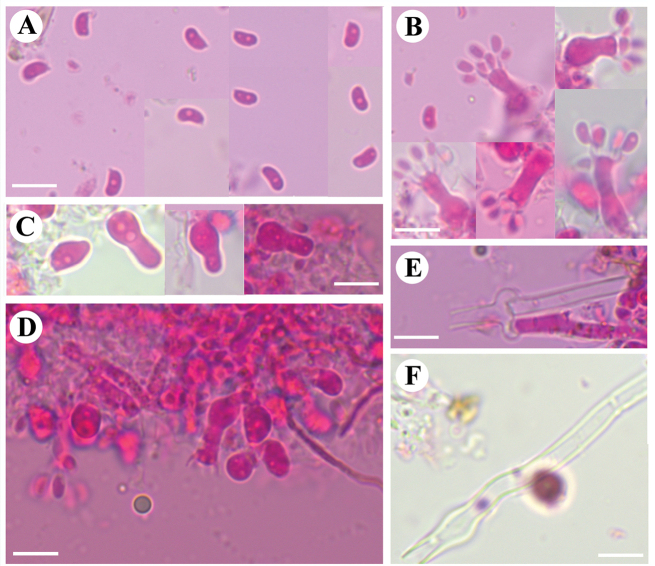
Microscopic structures of *Burgellaalbofarinacea* (holotype): basidiospores (**A**), basidia (**B**), basidioles (**C**), a section of hymenium (**D**), generative hyphae (**E, F**). Scale bars: 10 µm (**A–F**).

Basidiospores ellipsoid to allantoid, colorless, thin-walled, smooth, with oil drop, IKI–, CB–, (4–)4.5–6.5(–7) × 2–4 μm, L = 5.34 µm, W = 2.83 µm, Q = 1.79–1.97 μm (n = 120/4).

##### Additional specimens examined (paratypes).

China • Yunnan Province, Zhaotong, Yiliang County, Longan Town, GPS coordinates: 27°73′N, 104°16′E, altitude: 1550 m asl., on the fallen branch of angiosperm, leg. C.L. Zhao, 27 August 2023, CLZhao 32026, CLZhao 31855 (SWFC) • Zhaotong, Yiliang County, Xiaocaoba Town, GPS coordinates: 27°26′N, 104°26′E, altitude: 2225 m asl., on the fallen branch of angiosperm, leg. C.L. Zhao, 28 August 2023, CLZhao 32468 (SWFC).

#### ﻿ Burgella fissurata

Taxon classificationFungiCantharellalesHydnaceae

Q. Zhou & C.L. Zhao sp. nov.

F2CDDF76-4168-598C-B5CB-B26A636EE51A

857297

Figs 6 , 7 , 8

##### Holotype.

China • Yunnan Province: Dehong, Yingjiang County, Tongbiguan provincial nature reserve, GPS coordinates: 24°30′N, 097°30′E, altitude: 1300 m asl., on the fallen branch of angiosperm, leg. C.L. Zhao, 19 July 2023, CLZhao 30212 (SWFC).

##### Etymology.

*fissurata* (Lat.): refers to the cracking hymenial surface of the type specimens.

##### Description.

Basidiomata annual, resupinate, adnate, pruinose, hypochnoid, without odor or taste when fresh, up to 10.2 cm long, 1 cm wide, 50–100 µm thick. Hymenial surface smooth, cracked, white when fresh, turning to pale cream upon drying. Sterile margin thin, white, thinning out, up to 1 mm wide.

**Figure 6.**
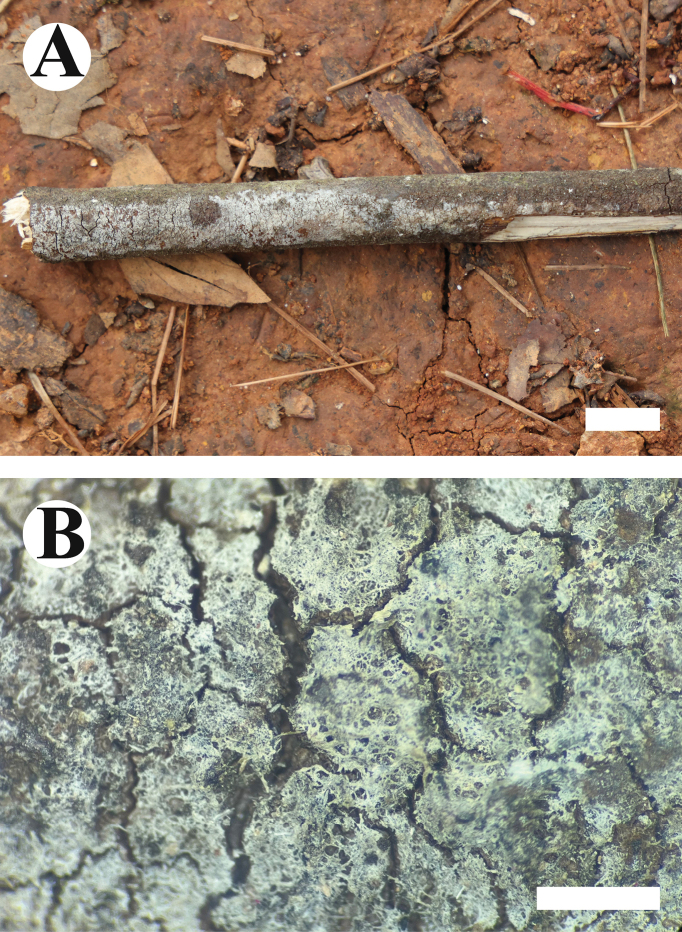
Basidiomata of *Burgellafissurata* (holotype). Scale bars: 1 cm (**A**); 1 mm (**B**).

Hyphal system monomitic, generative hyphae with clamp connections, colorless, thin-walled, frequently branched, interwoven, 2–4 μm in diameter; IKI–, CB–, tissues unchanged in KOH.

**Figure 7.**
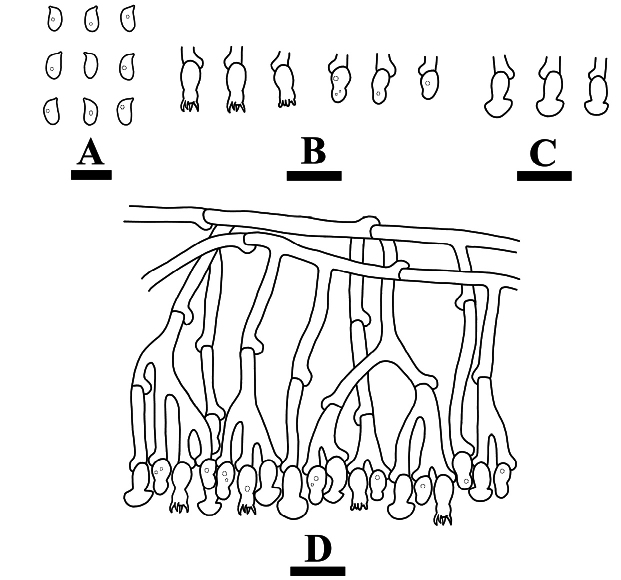
Microscopic structures of *Burgellafissurata* (holotype): basidiospores (**A**), basidia and basidioles (**B**), umbrella-shaped cystidia (**C**), a section of hymenium (**D**). Scale bars: 5 µm (**A**); 10 µm (**B, C**).

Cystidia umbrella-shaped, colorless, thin-walled, smooth, 5.5–10 × 4–6 µm; cystidioles absent. Basidia urniform, with a median constriction, thin-walled, with 4 sterigmata and a basal clamp connection, 6–11.5 × 2–4.5 μm; basidioles abundant, in shape similar to basidia, but slightly smaller.

**Figure 8.**
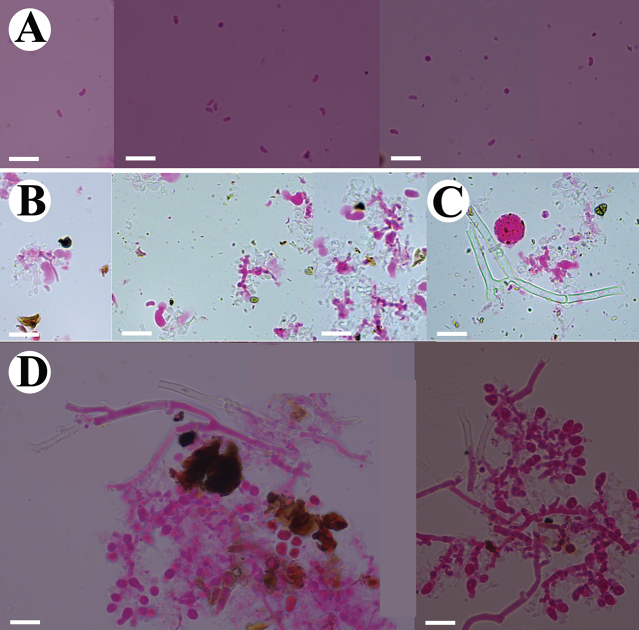
Microscopic structures of *Burgellafissurata* (holotype): basidiospores (**A**), basidia and basidioles; umbrella-shaped cystidia (**B**), generative hyphae (**C**), a section of hymenium (**D**). Scale bars: 10 µm (**A–D**).

Basidiospores narrowly ellipsoid, colorless, thin-walled, smooth, IKI–, CB–, (2.5–)3–4 × 1–2 μm, L = 3.36 µm, W = 1.63 µm, Q = 2.06 μm (n = 30/1).

#### ﻿ Burgoa wumengshanensis

Taxon classificationFungiCantharellalesHydnaceae

Q. Zhou & C.L. Zhao sp. nov.

A66E80E0-33C3-56B0-B936-F0A92C534D8C

857298

Figs 9 , 10 , 11

##### Holotype.

China • Yunnan Province: Zhaotong, Yiliang County, Luozehe Town, Lijiaping Village, Wumengshan National Nature Reserve, GPS coordinates: 27°29′N, 103°55′E, altitude: 1900 m asl., on the fallen branch of angiosperm, leg. C.L. Zhao, 19 September 2023, CLZhao 33227 (SWFC).

##### Etymology.

*wumengshanensis* (Lat.): refers to the locality, Wumengshan National Natural Reserve, of the type specimens.

**Figure 9.**
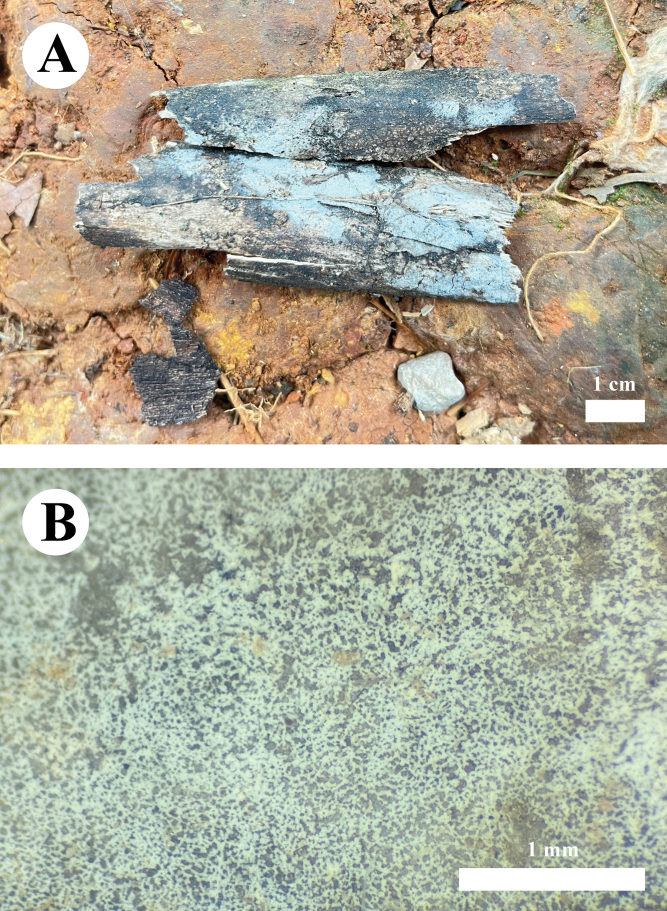
Basidiomata of *Burgoawumengshanensis* (holotype). Scale bars: 1 cm (**A**); 1 mm (**B**)

##### Description.

Basidiomata annual, resupinate, adnate, pellicular, pruinose upon drying, without odor or taste when fresh, up to 7.4 cm long, 2.1 cm wide, 40–90 µm thick. Hymenial surface smooth, white when fresh, turning to pale cream upon drying. Sterile margin thin, white, up to 1 mm wide.

**Figure 10.**
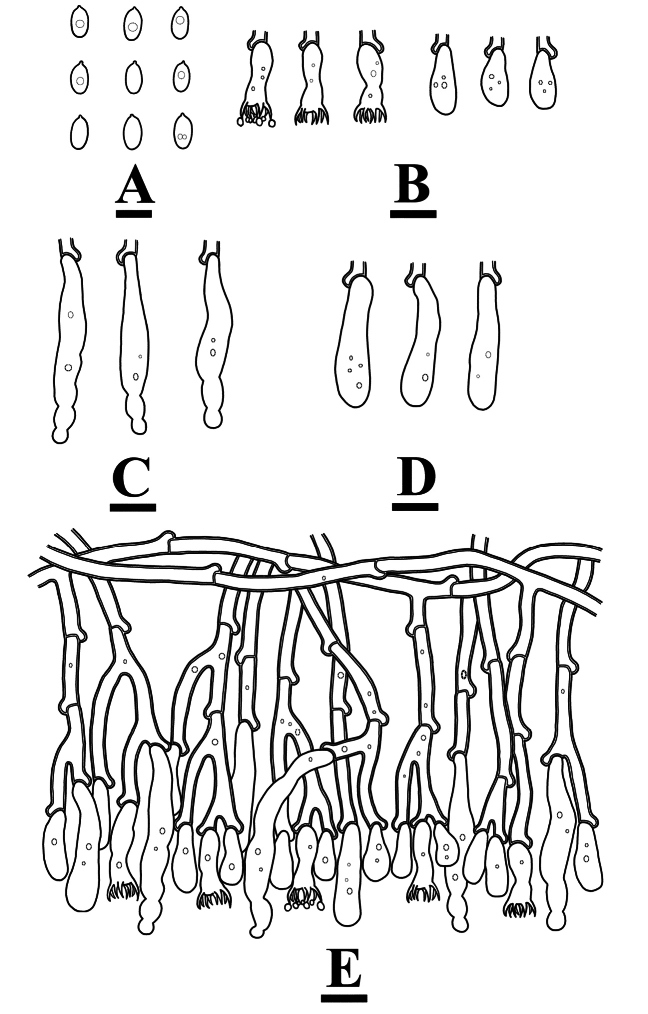
Microscopic structures of *Burgoawumengshanensis* (holotype) basidiospores (**A**), basidia and basidioles (**B**), schizopapillate cystidia (**C**), clavate cystidioles (**D**), a section of hymenium (**E**). Scale bars: 5 µm (**A**), 10 µm (**B, E**).

Hyphal system monomitic, generative hyphae with clamp connections, sometimes with oily contents, colorless, slightly thick-walled, frequently branched, interwoven, 3.5–5 µm in diameter; IKI–, CB–, tissues unchanged in KOH.

**Figure 11.**
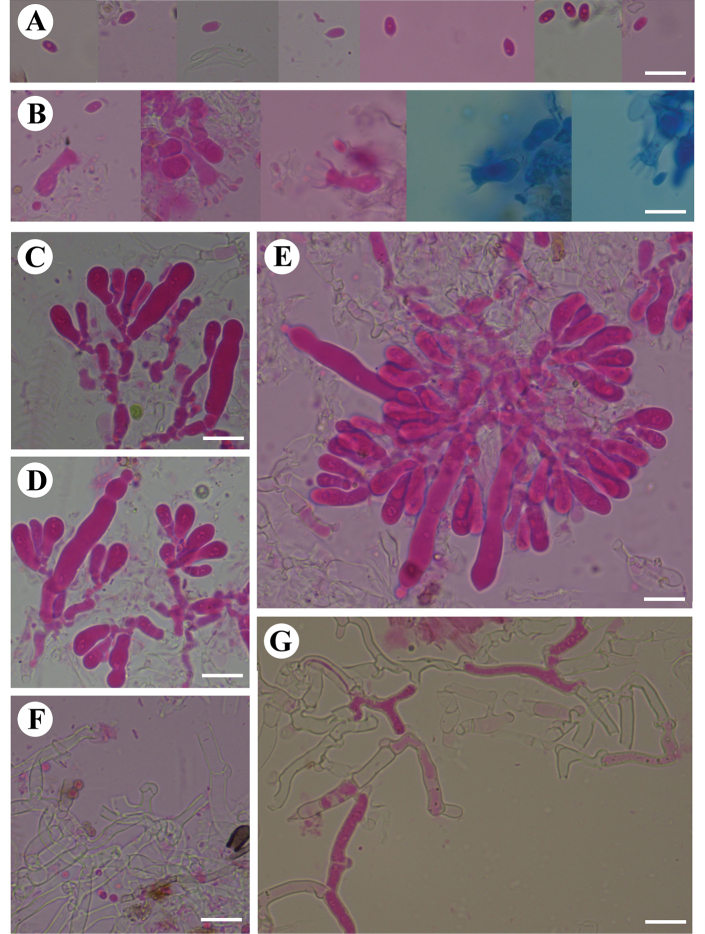
Microscopic structures of *Burgoawumengshanensis* (holotype) basidiospores (**A**), basidia (**B**), clavate cystidioles **(C**), schizopapillate cystidia (**D**), a section of hymenium (**E**), generative hyphae (**F, G**). Scale bars: 10 µm (**A–G**).

Cystidia schizopapillate, colorless, thin-walled, smooth, 30.5–49 × 5.5–8 µm; cystidioles clavate, colorless, thin-walled, smooth, 22–29.5 × 5–8 µm. Basidia urniform, with a median constriction, slightly thick-walled, with 8 sterigmata and a basal clamp connection, 12–20 × 3.5–6 µm; basidioles abundant, in shape similar to basidia, but slightly smaller.

Basidiospores ellipsoid, colorless, thin-walled, smooth, with oil drop, IKI–, CB–, (3.5)–4–5.5(–6) × 2–3.5 µm, L = 4.5 µm, W = 2.78 µm, Q = 1.62 (n = 30/1).

#### ﻿ Clavuliella

Taxon classificationFungiCantharellalesHydnaceae

Q. Zhou & C.L. Zhao gen. nov.

DDF9D9F6-E815-53E6-BDDA-1598E2418025

858532

##### Type species.

*Clavuliellasinensis* Q. Zhou & C.L. Zhao, sp. nov.

##### Etymology.

*Clavuliella* (Lat.): refers to the related genus *Clavulina*.

##### Description.

Basidiomata annual, coralloid, gregarious to caespitose clusters, greyish white to light grey when fresh, turning to dark grey upon drying; with sharply acuminate or cristate tips. Hyphal system monomitic, generative hyphae simple-septa, colorless, slightly thick-walled, frequently branched, interwoven. Cystidia and cystidioles absent. Basidia cylindrical, with a median constriction, with 2 sterigmata and a basal simple-septum, with oily contents. Basidiospores subglobose to broadly ellipsoid, colorless, thin-walled, smooth, with oil drop, IKI–, CB–.

##### Notes.

In our phylogenetical analyses (Fig. 1), *Clavuliella* was identified as a monophyletic group, typified by *C.sinensis.* The new genus *Clavuliella* falls within the family Hydnaceae (Cantharellales) and is closely related to *Clavulina. Clavulina* is distinguished from *Clavuliella* by its clavarioid to coralloid, simple or branched basidiomata with amphigenous hymenia, cylindrical to subclavate basidia with two or more cornuted sterigmata (Schröter 1888; Uehling et al. 2012; He et al. 2019; Gao et al. 2024).

*Clavuliella* resembles *Clavulina* in sharing coralloid basidiomata, subglobose, thin-walled basidiospores. However, *Clavuliella* differs from *Clavulina* by cylindrical basidia, with a median constriction, slightly thick-walled, with 2 sterigmata and a basal simple-septum, with oily contents and simple-septa generative hyphae. In this study, *Clavuliella* originating from the subtropical regions, suggests the possibility of discovering new corticioid taxa through further investigations and molecular analyses.

#### ﻿ Clavuliella sinensis

Taxon classificationFungiCantharellalesHydnaceae

Q. Zhou & C.L. Zhao sp. nov.

FF1BAA6A-D40A-5FF2-97BD-0F8FA8756525

858533

Figs 12 , 13 , 14

##### Holotype.

China • Guizhou Province: Guiyang, Qianlingshan Forest Park, GPS coordinates: 26°36′N, 106°41′E, altitude: 1396 m asl., on the ground, leg. C.L. Zhao, 21 August 2023, CLZhao 31231 (SWFC).

##### Etymology.

*sinensis* (Lat.): refers to the type locality (China).

##### Description.

Basidiomata annual, coralloid, gregarious to caespitose clusters, 0.6–1.5 cm tall, 0.62–1.6 cm wide, frequently branched 2–3 times, forming dichotomous branches at the apices, without odor or taste, soft when fresh, becoming brittle upon drying, usually lacking obvious basal mycelium; greyish white to light grey when fresh, turning to dark grey upon drying; with sharply acuminate or cristate tips.

**Figure 12.**
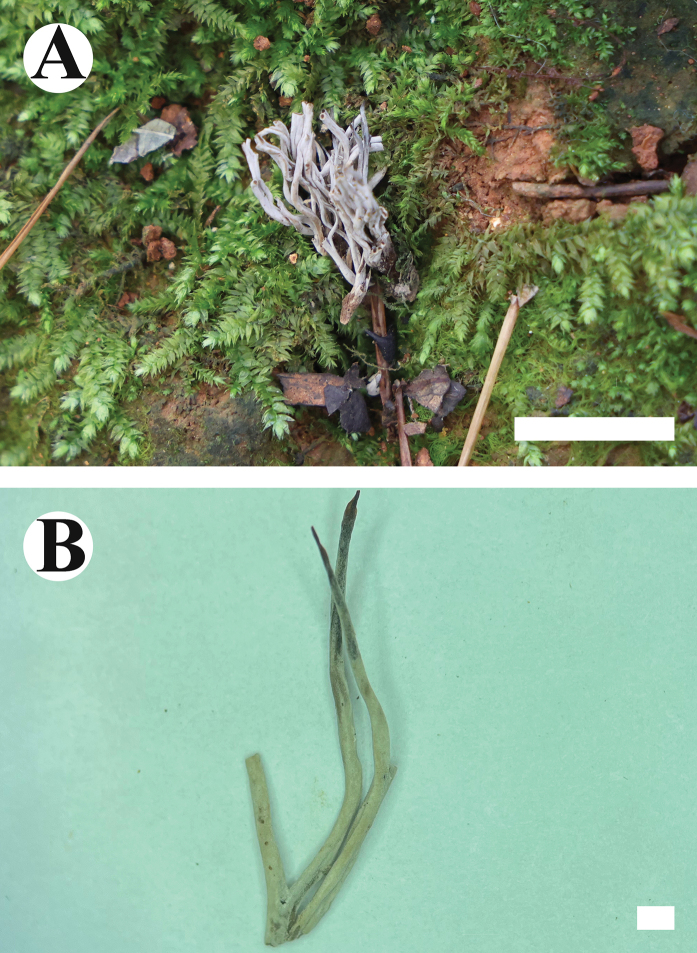
Basidiomata of *Clavuliellasinensis* (holotype). Scale bars: 1 cm (**A**); 1 mm (**B**).

Hyphal system monomitic, generative hyphae simple-septa, colorless, slightly thick-walled, frequently branched, interwoven, 4–10.5 μm in diameter; IKI–, CB–, tissues unchanged in KOH.

**Figure 13.**
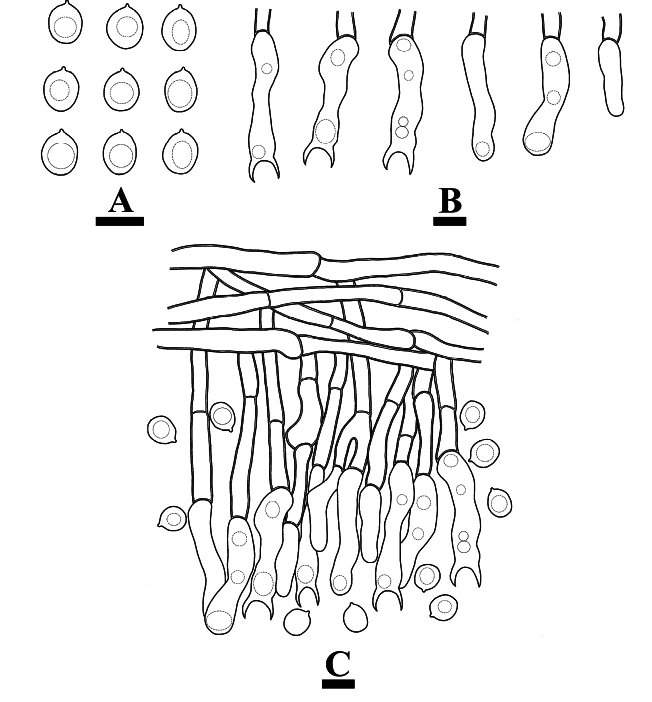
Microscopic structures of *Clavuliellasinensis* (holotype): basidiospores **(A**), basidia and basidioles (**B**), a section of hymenium (**C**). Scale bars: 5 µm (**A**); 10 µm (**B, C**).

Cystidia and cystidioles absent. Basidia cylindrical, with a median constriction, slightly thick-walled, with 2 sterigmata and a basal simple-septum, with oily contents, 18.5–43 × 6–9 μm; basidioles abundant, in shape similar to basidia, but slightly smaller.

**Figure 14.**
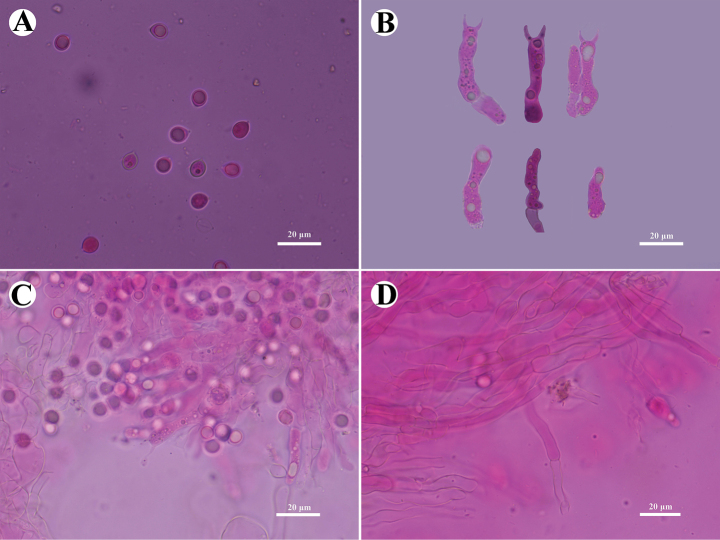
Microscopic structures of *Clavuliellasinensis* (holotype): basidiospores (**A**), basidia and basidioles (**B**), a section of hymenium (**C**), generative hyphae (**D**). Scale bars: 10 µm (**A–D**).

Basidiospores subglobose to broadly ellipsoid, colorless, thin-walled, smooth, with oil drop, IKI–, CB–, (7–)7.5–9.5(–10) × 6.5–8(–8.5) μm, L = 8.52 µm, W = 7.51 µm, Q = 1.13 μm (n = 30/1).

#### ﻿ Sistotrema sinense

Taxon classificationFungiCantharellalesHydnaceae

Q. Zhou & C.L. Zhao sp. nov.

D0864714-BA6E-5CEA-861F-C1A84E938B0A

857299

Figs 15 , 16 , 17

##### Holotype.

China • Yunnan Province: Dali, Weishan County, Qinghua Town, GPS coordinates: 25°01′N, 100°22′E, altitude: 2071.6 m asl., on the fallen branch of angiosperm, leg. C.L. Zhao, 18 October 2022, CLZhao 24876 (SWFC).

**Figure 15.**
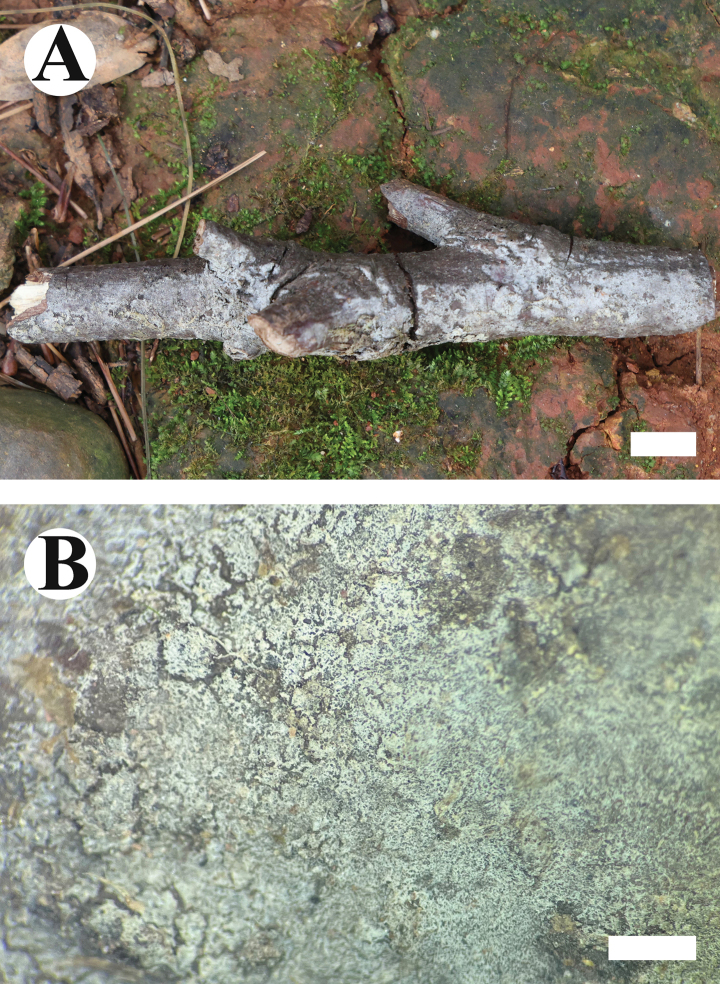
Basidiomata of *Sistotremasinense* (holotype). Scale bars: 1 cm (**A**); 1 mm (**B**).

##### Etymology.

*sinense* (Lat.): refers to the type locality (China).

##### Description.

Basidiomata annual, resupinate, adnate, soft coriaceous when fresh, becoming coriaceous upon drying, without odor or taste when fresh, up to 11 cm long, 2.5 cm wide, 50–100 µm thick. Hymenial surface smooth, white when fresh, turning to white to incanus upon drying. Sterile margin thin, white, thinning out, up to 1 mm wide.

**Figure 16.**
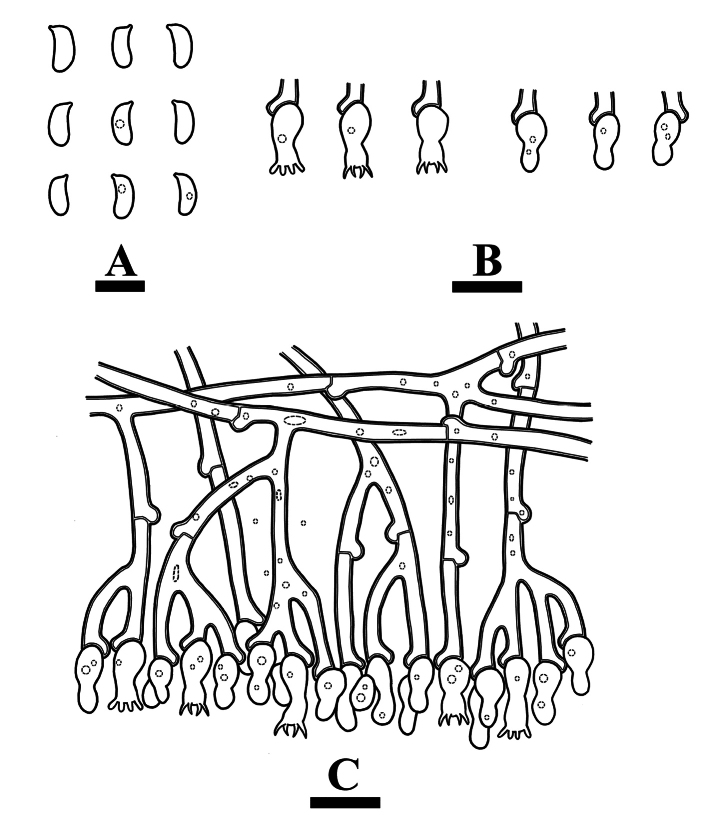
Microscopic structures of *Sistotremasinense* (holotype): basidiospores (**A**), basidia and basidioles (**B**), a section of hymenium (**C**). Scale bars: 5 µm (**A**); 10 µm (**B, C**).

Hyphal system monomitic, generative hyphae with clamp connections, often and characteristically with oil content, colorless, slightly thick-walled, frequently branched, interwoven, 2–4 μm in diameter; IKI–, CB–, tissues unchanged in KOH.

**Figure 17.**
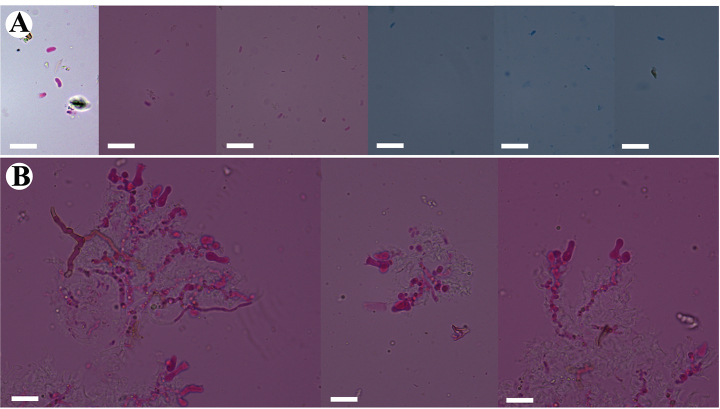
Microscopic structures of *Sistotremasinense* (holotype): basidiospores (**A**), a section of hymenium (**B**). Scale bars: 10 µm (**A, B**).

Cystidia and cystidioles absent. Basidia suburniform to urniform, slightly thick-walled, with 4 sterigmata and a basal clamp connection, 8–13.5 × 3–5 μm; basidioles abundant, in shape similar to basidia, but slightly smaller.

Basidiospores suballantoid to allantoid, colorless, thin-walled, smooth, IKI–, CB–, 3–4.5(–5) × (1–)1.5–2.5 μm L = 3.8 µm, W = 1.72 µm, Q = 2.21 μm (n = 30/1).

## ﻿Discussion

In recent years, many taxa of wood-inhabiting fungi have been continuously reported and recorded all over the world, including in the genus *Burgella*, *Burgoa*, and *Sistotrema* (Diederich et al. 2014; Koukol and Kubátová 2015; Cai and Zhao 2023; Dong et al. 2024b; Yuan et al. 2024; Zhang et al. 2024; Yang et al. 2025). Several previous studies, based on ITS+nLSU sequence data, confirmed phylogenetic relationships, in which the genus *Burgella*, *Burgoa*, *Clavuliella*, and *Sistotrema* are nested in the order Cantharellales (Diederich et al. 2014; Koukol and Kubátová 2015).

Phylogenetically, based on the multiple loci in the family Hydnaceae, four genera; *Burgella*, *Burgoa*, *Clavuliella*, and *Sistotrema* were located in this family (Zhou and Qin 2013; Vu et al. 2019; Masumoto and Degawa 2020; Sugawara et al. 2022; Campo et al. 2023; Cai and Zhao 2023). In the present study, based on the phylogram inferred from the ITS+nLSU data (Fig. 1), two new species were grouped into the genus *Burgella*, in which *B.albofarinacea* was closely related to *B.flavoparmeliae* and *B.fissurata* was grouped with *B.lutea*. The phylogram based on inferences from the ITS and nLSU data (Fig. 1) showed that *B.wumengshanensis* clustered into the genus *Burgoa*, in which it was grouped closely with *B.anomala* and *B.verzuoliana*. The phylogram based on inferences from the ITS and nLSU data (Fig. 1) showed that *Clavuliella* was identified as a monophyletic group, typified by *C.sinensis*, in which it was grouped closely with *C.minor* X.X. Huang & L.H. Qiu, *C.cristata*, and *C.iris* Loizides, Bellanger & P.-A. Moreau. The phylogenetic tree (Fig. 2), inferred from the ITS+nLSU sequences, highlighted that *Sistotremasinense* was grouped closely with *S.brinkmannii* and *S.farinaceum*.

Morphologically, *Burgellaflavoparmeliae*, *B.lutea* and *B.fissurata* are similar to *B.albofarinacea* by having the generative hyphae with septa or with clamp connections. (Diederich and Lawrey 2007). However, *B.flavoparmeliae* differs from *B.albofarinacea* by the irregularly shaped, coralloid, orange agglomerations of bulbils and generative hyphae with septa or without clamp connections, with both morphologies present on the same hyphae at neighboring septa (Diederich and Lawrey 2007). *B.lutea* is distinguished from *B.fissurata* by the superficial, yellow to orange-yellow, roundish bulbils (Diederich et al. 2014). *B.fissurata* is distinguishable from *B.albofarinacea* by the pruinose hypochnoid hymenial surface, with umbrella-shaped cystidia, basidia with 4 sterigmata, and its smaller basidia (6–11.5 × 2–4.5 μm; Diederich and Lawrey 2007; Koukol and Kubátová 2015; Kiyuna et al. 2015).

*Burgellaflavoparmeliae* is separated from *B.fissurata* by the irregularly shaped, coralloid, orange agglomerations of bulbils and generative hyphae with septa or without clamp connections, both situations present on the same hyphae at neighbouring septa (Diederich and Lawrey 2007). *B.lutea* is distinguished from *B.fissurata* by the superficial, yellow to orange-yellow, roundish bulbils (Diederich et al. 2014). *B.albofarinacea* differs from *B.fissurata* by the pellicular, coriaceous hymenial surface, bigger basidia with 8 sterigmata and bigger basidiospores (Cai and Zhao 2023).

Morphologically, *Burgoaanomala* and *B.verzuoliana* are similar to *B.wumengshanensis* by having the generative hyphae with clamp connections (Koukol and Kubátová 2015). However, *B.anomala* is distinguishable from *B.wumengshanensis* by having spherical bulbils, and hyaline to pale brown generative hyphae, thin-walled, thinner (2–5 μm) in diameter (Koukol and Kubátová 2015); *B.verzuoliana* is distinguished from *B.wumengshanensis* by having spherical bulbils (Diederich and Lawrey 2007).

Morphologically, *Clavulinacristata*, *C.griseoviolacea* Yue Gao, Hao Zhou, & C.L. Hou, and *C.pallida* Yue Gao, Hao Zhou & C.L. Hou are similar to *Clavuliellasinensis* by having clavarioid to coralloid basidiomata and guttulate basidiospores (Uehling et al. 2012; Gao et al. 2024). However, *Clavulinacristata* is separated from *Clavuliellasinensis* by having cylindrical to subclavate basidia with two or more cornuted sterigmata (Gao et al. 2024); *Clavulinagriseoviolacea* differs from *Clavuliellasinensis* by having gray to dark grayish violet basidiomata with a white stipe, hyphae with clamp connections, and smaller basidiospores (6.5–8.0 × 6.2–7.2 μm; Crous et al. 2014); *Clavulinapallida* is distinguishable from *Clavuliellasinensis* by having solitary or scattered basidiomata, generative hyphae clamp connections, and longer basidia (34.2–48.5 × 4.8–6.3 μm; Gao et al. 2024).

Morphologically, *Sistotremadiademiferum* (Bourdot & Galzin) Donk, *S.coroniferum* (Höhn. & Litsch.) Donk and *S.hispanicum* M. Dueñas, Ryvarden & Tellería are similar to *S.sinense* by having the urniform basidia and basal hyphae with clamp connections (Bernicchia and Gorjón 2010). However, *S.diademiferum* is separated from *S.sinense* by the smooth, porulose hymenophore, larger basidia with 6 sterigmata (15–20 × 5–7 μm), and ovoid to subglobose basidiospores (Bernicchia and Gorjón 2010). *S.coroniferum* is distinguishable from *S.sinense* by the smooth hymenophore, with gloeocystidia, basidia with 6 sterigmata, and longer subcylindrical basidiospores (5–6 × 2–2.5 µm; Bernicchia and Gorjón 2010). *S.hispanicum* differs from *S.sinense* by the whitish to yellow hymenial surface and bigger narrowly ellipsoid to subreniform basidiospores (5.5–6 × 3–4 μm; Bernicchia and Gorjón 2010).

As wood-inhabiting fungi efficiently degrade lignocellulose in wood, they play a crucial ecological role in material recycling and energy flow in forest ecosystems, as well as playing a major economic role (Sugawara et al. 2022; Zhang et al. 2022; Bondartseva and Zmitrovich 2023; Campo et al. 2023; Cui et al. 2019; Gao et al. 2024; Liu et al. 2023a, b; Sun et al. 2020; Sun et al. 2022; Ji et al. 2022). Wood-inhabiting fungi are an extensively studied group of Basidiomycota, but their diversity is still not well known in China, and many recently described taxa in this ecological group have been discovered from China (Sugawara et al. 2022; Cai and Zhao 2023; Wang et al. 2023; Wang et al. 2024; Wu et al. 2022; Yuan et al. 2023; Zhao et al. 2024). Four new species and a new genus, from the Yunnan and Guizhou Provinces of China, serve as examples of the understudied fungal diversity present in the P.R. of China.. On a wider scale, this study enriches our knowledge on the diversity of wood-inhabiting fungi worldwide.

## Supplementary Material

XML Treatment for Burgella albofarinacea

XML Treatment for Burgella fissurata

XML Treatment for Burgoa wumengshanensis

XML Treatment for Clavuliella

XML Treatment for Clavuliella sinensis

XML Treatment for Sistotrema sinense

## ﻿Additional information

### Author ORCIDs

Qian Zhou https://orcid.org/0009-0007-0845-2115

Chengbin Qian https://orcid.org/0009-0003-5329-1016

Chuyun Zhang https://orcid.org/0009-0008-8239-8492

Qidong Su https://orcid.org/0009-0000-1521-7606

Yiliang Li https://orcid.org/0009-0009-9599-256X

Shihui Zhang https://orcid.org/0009-0008-3309-4214

Nian Mu https://orcid.org/0009-0009-1316-8658

Taimin Xu https://orcid.org/0000-0002-5230-4140

Hongmin Zhou https://orcid.org/0000-0002-0724-5815

Changlin Zhao https://orcid.org/0000-0002-8668-1075

### Data availability

All of the data that support the findings of this study are available in the main text.
